# Chemical Structures of 4-Oxo-Flavonoids in Relation to Inhibition of Oxidized Low-Density Lipoprotein (LDL)-Induced Vascular Endothelial Dysfunction

**DOI:** 10.3390/ijms12095471

**Published:** 2011-08-26

**Authors:** Long Yi, Xin Jin, Chun-Ye Chen, Yu-Jie Fu, Ting Zhang, Hui Chang, Yong Zhou, Jun-Dong Zhu, Qian-Yong Zhang, Man-Tian Mi

**Affiliations:** Research Center for Nutrition and Food Safety, Chongqing Key Laboratory of Nutrition and Food Safety, Institute of Military Preventive Medicine, Third Military Medical University, Chongqing 400038, China; E-Mails: longgyin8341@hotmail.com (L.Y.); jinxin41@hotmail.com (X.J.); xiguameimei-ye@163.com (C.-Y.C.); fuyujie2011@hotmail.com (Y.-J.F.); zhangting20091@hotmail.com (T.Z.); changhui1998@hotmail.com (H.C.); zhouyong19621@hotmail.com (Y.Z.); zhangqianyong2011@hotmail.com (Q.-Y.Z)

**Keywords:** flavonoids, endothelial dysfunction, oxidized low-density lipoprotein, structure-activity analysis, atherosclerosis, reactive oxygen species

## Abstract

Vascular endothelial dysfunction induced by oxidative stress has been demonstrated to be the initiation step of atherosclerosis (AS), and flavonoids may play an important role in AS prevention and therapy. Twenty-three flavonoids categorized into flavones, flavonols, isoflavones, and flavanones, all with 4-oxo-pyronenucleus, were examined for what structural characteristics are required for the inhibitory effects on endothelial dysfunction induced by oxidized low-density lipoprotein (oxLDL). Human vascular endothelial cells EA.hy926 were pretreated with different 4-oxo-flavonoids for 2 hs, and then exposed to oxLDL for another 24 hs. Cell viability and the level of malondialdehyde (MDA), nitric oxide (NO) and soluble intercellular adhesion molecule-1 (sICAM-1) were measured, respectively. Then, correlation analysis and paired comparison were used to analyze the structure–activity relationships. Significant correlations were observed between the number of −OH moieties in total or in B-ring and the inhibitory effectson endothelial dysfunction. Furthermore, 3′,4′-ortho-dihydroxyl on B-ring, 3-hydroxyl on C-ring and 2,3-double bondwere correlated closely to the inhibitory effects of flavonolson cell viability decrease and lipid peroxidation. 5,7-meta-dihydroxyl group on A-ring was crucial for the anti-inflammatory effects of flavones and isoflavones in endothelial cells. Moreover, the substituted position of B-ring on C3 rather than C2 was important for NO release. Additionally, hydroxylation at C6 position significantly attenuated the inhibitory effects of 4-oxo-flavonoids on endothelial dysfunction. Our findings indicated that the effective agents in inhibiting endothelial dysfunction include myricetin, quercetin, luteolin, apigenin, genistein and daidzein. Our work might provide some evidence for AS prevention and a strategy for the design of novel AS preventive agents.

## 1. Introduction

Atherosclerosis (AS) is a multifactorial disease of the vessel wall involving lipid accumulation, thrombogenic components, cell death and inflammatory responses in the arterial wall, causing heart disease and stroke [1]. High plasma level of low-density lipoprotein (LDL) is closely correlated with accelerated atherogenesis [2,3]. LDL could be oxidatively modified to be oxidized LDL (oxLDL) by transition metal ions such as copper ion (Cu^2+^), or by inorganic oxidants such as H_2_O_2_. oxLDL is more important than native-LDL for atherogenesis, mainly leading to vascular endothelial dysfunction, foam cell formation and smooth cell proliferation [4]. Endothelial dysfunction is characterized by a shift of the actions of the endothelium toward increased lipid oxidation, reduced vasodilation, a proinflammatory state and prothrombic properties. Therefore, endothelial dysfunction induced by oxLDL has been known to be a key event in the development of AS and predates clinically obvious vascular pathology by many years [5,6].

**Figure 1:**
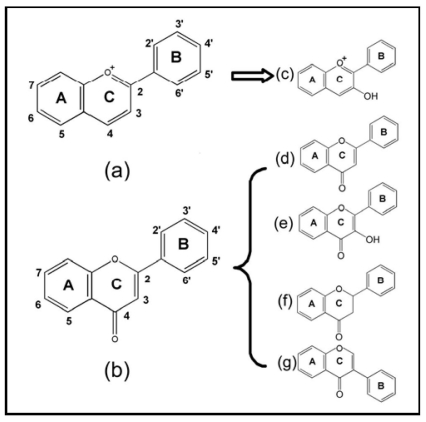
The classification and structure of the flavonoids: (**a**) flavan nucleus; (**b**) 4-oxo-flavonoid nucleus; (**c**) anthocyanidins; (**d**) flavones; (**e**) flavonols; (**f**) flavanones and (**g**) isoflavones.

Flavonoids are important phytonutrient components distributed in a wide range of fruits, vegetables, nuts and beverages, including wine and tea. Roughly speaking, there are more than 4000 types of flavonoids. The structure of the flavonoids is based on the flavonoid nucleus (Figure 1a,b), which consists of three phenolic rings referred to as the A, B, and C rings. The benzene ring A is condensed with a six-member ring (C-ring), which in the 2-position carries a phenyl benzene ring (B-ring) as a substituent. Ring C may be a heterocyclic pyran, which yields anthocyanins (Figure 1c), or 4-oxo-pyrone, which yields flavones (Figure 1d), flavonols (Figure 1e), flavanones (Figure 1f) and isoflavones (Figure 1g) [7]. Epidemiological studies have revealed an inverse association between dietary flavonoids intake and the occurrence of cardiovascular disease, such as AS [8]. This raises the possibility that flavonoids may act as a lead compound for the development of therapeutic agents, which can be used in AS prevention and therapy.

Since different flavonoids might have different roles in endothelial cells, the structure–activity relationships of flavonoids have become the subject of many investigations [9,10]. Certain flavonoids such as quercetin are potent in scavenging radicals, while genistein could ameliorate endothelium-dependent vasorelaxation [11,12]. In our previous study, we firstly reported the differences of anthocyanins, one subclass of flavonoids with a flavan nucleus, in the inhibition of oxLDL-induced endothelial injury, and clarified the main structural requirements for the inhibitory effects [13]. However, there are large numbers of flavonoids, in particular flavonoids with 4-oxo-pyronenucleus widely distributed in the plant foods. Few investigations have examined what structural characteristics are required for the inhibitory effects on endothelial dysfunction. In the present study, we screened a total of 23 flavonoids (Table 1) with a 4-oxo-pyrone nucleus, categorized into flavones, flavonols, flavanones and isoflavones, and compared the different effects on the cell viability, lipid oxidation, secretory function and inflammatory reaction in oxLDL-induced endothelial cells. Our work provides evidence that certain chemical structures of 4-oxo-flavonoids were crucial for the inhibitory effects on endothelial dysfunction.

## 2. Results

**Figure 2:**
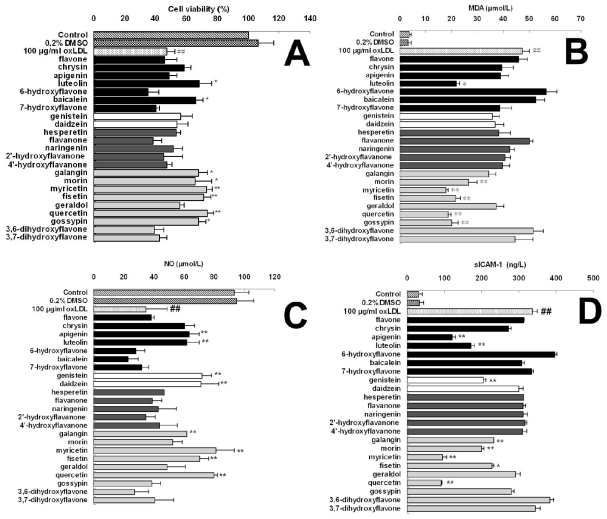
Effect of different 4-oxo-flavonoids of 40 μM on oxLDL-induced endothelial viability (**A**) and the level of MDA (**B**), NO (**C**) and sICAM-1 (**D**). EA.hy926 cells were pretreated with different 4-oxo-flavonoids of 40 μM, and exposed to oxLDL of 100 μg/mL for another 24 hs. Cell viability was measured by CCK-8 assay and the values were determined as the percentage of the control group. The level of MDA, NO, and sICAM-1 were determined by the corresponding detecting kits. All of the data are expressed as the means ± S.D. ^#^ *P* < 0.05, ^##^ *P* < 0.01, compared with the control group; * *P* < 0.05, ** *P* < 0.01, compared with the oxLDL group.

In this work, we selected 23 kinds of 4-oxo-flavonoids, including seven flavones, two isoflavones, five flavanones and nine flavonols, to evaluate their effects on oxLDL-induced endothelial dysfunction. The compounds name and structure characteristics are presented in Table 1 according to their subgroup classification. The inhibitory effects of flavonoids on oxLDL-induced endothelial dysfunction were evaluated by measuring cell viability and the level of malondialdehyde (MDA), nitric oxide (NO) and soluble intercellular adhesion molecule-1 (sICAM-1). Our results suggested that the inhibitory effects of different flavonoids on endothelial dysfunction were differential (Table 2 and Figure 2). All flavanones, structurally resembling flavones and flavonols containing a 2,3-double bond showed no notable effect on cell viability and the level of MDA, NO and sICAM-1 in oxLDL-induced endothelial cells at any treated concentration. However, most flavonols tested in our study, such as morin, myricetin, fisetin, and quercetin, demonstrated a significant effect in all measurements with concentration of more than 40μM. Two isoflavones, genistein and daidzein showed a dominantly enhanced effect on NO release, suggesting a particular effect on the endothelial secretory function and endothelium-dependent vasorelaxation. Luteolin and apigenin, belonging to the flavones, were prone to inhibit sICAM-1 generation and also promote NO release. Furthermore, our data showed that a few flavonoids, such as 6-hydroxyflavone and 3,6-dihydroxyflavone, exacerbated the oxLDL-induced endothelial dysfunction, suggesting a potentially negative effects on endothelial dysfunction. Additionally, significant correlations were observed between the effects of 4-oxo-flavonoids on oxLDL-induced cell viability and MDA generation (*r* = −0.812, *P* < 0.01), cell viability and NO release (*r* = 0.657, *P* < 0.01), cell viability and sICAM-1 (*r* = −0.677, *P* < 0.01), MDA generation and NO release (*r* = −0.743, *P* < 0.01), MDA generation and sICAM-1 (*r* = 0.717, *P* < 0.01); NO release and sICAM-1 (*r* = 0.810, *P* < 0.01), respectively.

**Figure 3:**
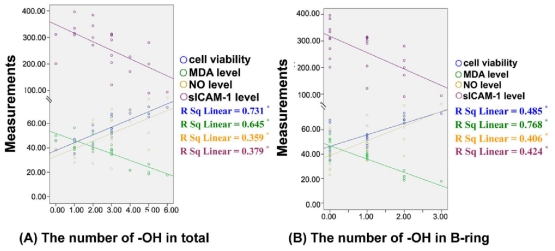
Correlation between the number of −OH groups of 4-oxo-flavonoids and the inhibitory effects on oxLDL-induced endothelial dysfunction. In the scatter plot, the X axis (horizontal axis) represents the number of −OH groups of flavonoids in total (**A**) or in B-ring; and (**B**), and the Y axis (vertical axis) represents the measurements of cell viability, MDA level NO level and sICAM-1 level, respectively. The overlay scatter plots represent 23 flavonoids in different experiments, respectively. Data were analyzed by Pearson’s correlation coefficient (*r*) and Fisher’s r to z (*p*) about 23 flavonoids. * *P* < 0.01.

We performed the correlation analysis and paired comparison to elucidate the structure–activity relationship of 4-oxo-flavonoids in inhibiting endothelial dysfunction. Figure 3 shows that the number of −OH groups of the tested 4-oxo-flavonoids in total (Figure 3A) and in B-ring (Figure 3B) ranged from 0–6 and 0–3, respectively. Using Pearson’s Correlation Analysis, we found a significant correlation between the number of −OH moities in total and the effect of 4-oxo-flavonoids (at 40 μM) on cell viability (correlation coefficient square (*R^2^* = 0.731, *P* <0.01), MDA level (*R^2^* = 0.645, *P* < 0.01), NO level (*R^2^* = 0.359, *P* < 0.01) and sICAM-1 level (*R^2^* = 0.379, *P* < 0.01). Moreover, significant linear relationships were also observed between the number of −OH moieties in B-ring and the effect of 4-oxo-flavonoids on cell viability (*R^2^* = 0.485, *P* < 0.01) and the level of MDA (*R^2^* = 0.768, *P* < 0.01), NO (*R^2^* = 0.406, *P* < 0.01) and sICAM-1 (*R^2^* = 0.424, *P* < 0.01), respectively. The results suggested that the inhibitory effects of 4-oxo-flavonoids on the oxLDL-induced endothelial dysfunction were enhanced with the increase of −OH moieties in total or in B-ring, respectively.

**Figure 4:**
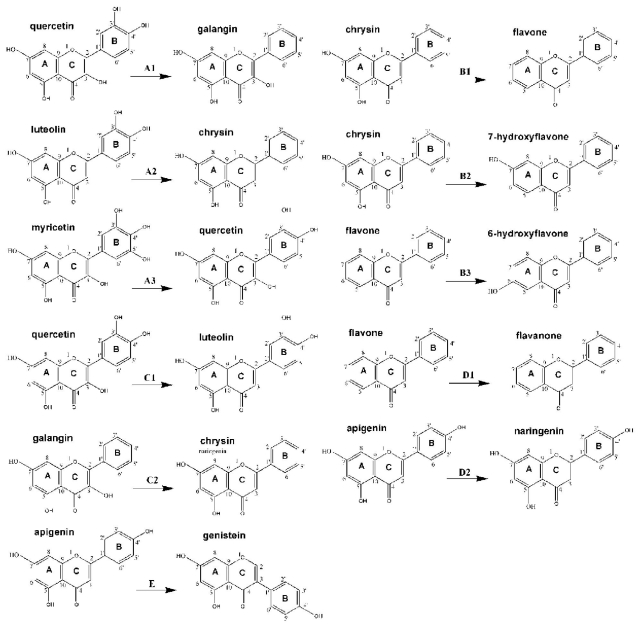
Effect of structural modificationsof 4-oxo-flavonoids on the inhibition of endothelial dysfunction by 3′,4′-ortho-dihydroxyl group (A1, A2 and A3), 5,7-meta-dihydroxyl group (B1, B2 and B3), 3-hydroxyl group (C1 and C2), 2,3-double bond (D1 and D2) and the substituted position of B-ring (E).

As shown in Table 3, we performed the paired comparison analysis to investigate the effect of different substitutions on ring-B (Table 3a), A (Table 3b) and C (Table 3c), the presence of 2,3-double bond (Table 3d) and the substituted position of B-ring (Table 3e) of 4-oxo-flavonoids on oxLDL-induced endothelial dysfunction. In general, the results demonstrated that a 3′,4′-ortho-dihydroxyl on B-ring, a 3-hydroxyl on C-ring and 2,3-double bond and a 5,7-meta-dihydroxyl on A-ring were all required for the inhibitory effects on endothelial dysfunction. However, the potential roles of these chemical characteristics of 4-oxo-flavonoids in relation to the inhibitory effects on endothelial dysfunction might be differential. Flavonols and flavones with a 3′,4′-ortho-dihydroxyl were much stronger in promoting cell viability and NO release as well as decreasing MDA and sICAM-1 generation, which implied the crucial roles of a 3′,4′-ortho-dihydroxyl group (Table 3b). Consistent with this finding, we found that quercetin (3′,4′,3,5,7-pentahydroxyflavone) was more efficient than galangin (3,5,7-trihydroxyflavone) (Figure 4A1), luteolin (3′,4′,5,7-tetrahydroxyflavone) was more active thanchrysin (5,7-dihydroxyflavone) (Figure 4A2), and fistin (3′,4′,3,7-tetrahydroxyl) suppressed the geraldol (3′-methoxyl, 4′,3,7-trihydroxyflavone) activity. Interestingly, the simultaneous presence of three hydroxyl groups at positions 3′, 4′ and 5′ in flavonols (e.g., myricetin *vs.* quercetin) did not significantly improve the inhibitory effect on endothelial injury (Figure 4A3). Moreover, favonols were superior to the flavanones with the same substituents in inhibiting endothelial dysfunction, due to the presence of 2,3-double bond (Table 3d, Figure 4D1,D2). Quercetin was much stronger than luteolin in decreasing MDA and sICAM-1 generation and enhancing NO release, suggesting the potential roles of 3-hydroxyl group (Table 3c, Figure 4C1,C2). Additionally, a 5,7-meta-dihydroxyl on A-ring might be correlated closely to the inhibitory effects on sICAM-1 generation (Table 3b, Figure 4B1,B2). However, adding another hydroxyl group in the A ring at 6-position significantly decreased the activity for chrysin, highlighting the importance of 5,7-meta-dihydroxyl groups in the anti-inflammatory activity (Table 3b and Figure 4D3). Therefore, fisetin with merely a −OH on C7 of A-ring, was less effective to quercetin. Interestingly, 6-hydroxyflavone and 3,6-dihydroxyflavone, both of which are structurally hydroxylated on C6 of A-ring, obviously aggravated endothelial dysfunction. Thus, baicalein, which is structurally identical to chrysin with a 5,7-meta-dihydroxyl but possess an another hydroxyl group on C6 position of A-ring, was ineffectual. Both isoflavones (genistein and daidzein) showed significant effects on stimulating the NO release. This result revealed that the substituted position of B-ring at C3 rather than that at C2 of B-ring greatly increased the effect of 4-oxo-flavonoids on endothelial secretory activity (Table 3e and Figure 4E).

Thus, we demonstrated that flavonols were prone to improve cell viability and inhibit lipid peroxidation induced by oxidative stress in endothelial cells correlated with the presence of a 3′,4′-ortho-dihydroxyl, a 3-hydroxyl and 2,3-double bond. While flavones with a 5,7-meta-dihydroxyl and isoflavones with a B-ring on C3 position were effectual to affect the inflammatory reaction and the secretory function in endothelial cells, respectively. Flavanone, without any typical characteristics above, showed no dominant effect. Furthermore, flavonoids such as myricetin, quercetin, genistein, daidzein, apigenin and luteolin were found with significant activity in inhibiting endothelial dysfunction.

## 3. Experimental Section

### 3.1. Reagents

Dulbecco’s modified Eagle’s medium (DMEM) and fetal bovine serum (FBS) were both purchased from Gibco Company (Gibco, NY, USA). Cell Counting Kit (CCK-8) was purchased from Dojindo Laboratories (Kumamoto, Japan). MDA and NO assay kits were both purchased from Nanjing Jiancheng Bioengineering Institute (Nanjing, China). A total of 23 4-oxo-flavonoids (Table 1), Enzyme-linked immunosorbent assay (ELISA) kit for sICAM-1 was obtained from Sigma-Aldrich (St. Louis, MO, USA). Flavonoids were dissolved in dimethyl sulfoxide (DMSO), with the final culture concentration of DMSO ≤ 0.2%.

### 3.2. Oxidation of Low-Density Lipoprotein

Human LDL was isolated from serum of 15 healthy donors (35.3 ± 6.28 years old; males/female: 8/7). This work was approved by Ethics Committee of Third Military Medical University, and each of the participants provided written informed consent. The oxLDL was prepared according to the method of Sternberg [14]. A 10 mg sample of LDL was dialyzed against Tris/NaCl buffer (50 mM Tris in 0.15 M NaCl, pH 8.0) to remove the EDTA. Afterwards, Tris/NaCl buffer was added to the dialyzed LDL to adjust the protein concentration to 30 mg/mL, then, 1 mL of 10 μM CuSO_4_ was added to 1 mL of dialyzed LDL. Oxidation at 37 °C for a period of 18 hs was followed by the addition of 300 μM EDTA to stop the reaction. The oxLDL was again dialyzed at 4 °C with Tris/NaCl buffer, filtered with a 0.22 μm filter, and stored in nitrogen at 4 °C. Oxidation was monitored by estimation of thiobarbituric acid reactive substances (TBARS) using malondialdehyde assay kit according to the manufacturer’s instructions.

### 3.3. Cell Culture

Human vascular endothelial cell line EA.hy926 was purchased from American Type Culture Collection (ATCC). The cells were maintained in DMEM medium containing 100 U/mL penicillin, 100 μg/mL streptomycin and 10% FBS at 37 °C under a humidified atmosphere with 5% CO_2_.

### 3.4. Cell Viability Measurement

CCK-8 detecting kit was used to measure cell viability as previously described [15]. EA.hy926 cells were seeded in 96-well microplate at a density of 1 × 10^4^ cells/well. The cells were pretreated for 2 hs with different 4-oxo-flavonoids at a series of concentration (5, 10, 20, 40 and 80 μM) and were then exposed to 100 μg/mL oxLDL for another 24 hs. Control and solvent control groups were treated with culture medium and medium with 0.2% DMSO, respectively. Subsequently, CCK-8 (20 μL/well) was added to the cells, and the plate was incubated at 37°C for 2 hs. Viable cells were counted by absorbance measurements with a monochromator microplate reader (SafireΠ, Tecan, Switzerland) at a wavelength of 450 nm. The optical density (OD) 450 value was proportional to the degree of cell viability. Cell viability in the control group was set as 100%, and the cell viability in the other groups was determined as the percentage of the control group.

### 3.5. Determination of MDA, NO and sICAM-1 Level

EA.hy926 cells were pretreated with different 4-oxo-flavonoids at a concentration of 40 μM for 2 hs, and then the cells were exposed to 100 μg/mL oxLDL for another 24 hs. The supernatant was collected from each well, and MDA, NO and sICAM-1 were measured with the corresponding assay kit according to the manufacturer’s instructions, respectively. Briefly, the MDA content in supernatants was measured by TBARS as previously described, and the optical density was measured at 532 nm [16]. MDA generated by acid hydrolysis of tetra-ethoxy-propane was used as a standard. NO in the supernatants was detected by measuring the concentration of nitrites (NO_2_ ^−^) and nitrates (NO_3_ ^−^), stable end products of NO at the wavelengths of 550 nm. Concentration of sICAM-1 in supernatants was determined by using human sICAM-1 ELISA kit. Absorbance of each well was determined within 30 mins using a Microplate Reader (Biotek, Winooski, VT) at 450nm and each sample value (ng/L) were calculated from the standard curve. All measurements in the control group were set as 100%, and the measurements in the other groups were determined as the percentage of the control group.

### 3.6. Structure-Activtity Relationship Analysis

Based on the measurements of cell viability, MDA level, NO level and sICAM-1 level, Pearson Correlation Assay was used to determine the correlation of all measurements. Meanwhile, paired comparison was carried out by Turkey-Kramer assay to elucidate the structural requirements of 4-oxo-flavonoids in relation to inhibition of endothelial dysfunction.

### 3.7. Statistical Analysis

The results were shown as mean ± S.D. Statistical analysis was performed by one-way analysis of variance. *P* < 0.05 was considered as statistically significant, and Tukey-Kramer was applied as *post-hoc* test if *P* < 0.05. The correlation between groups was evaluated by Pearson’s Correlation Analysis.

## 4. Discussion

**Figure 5:**
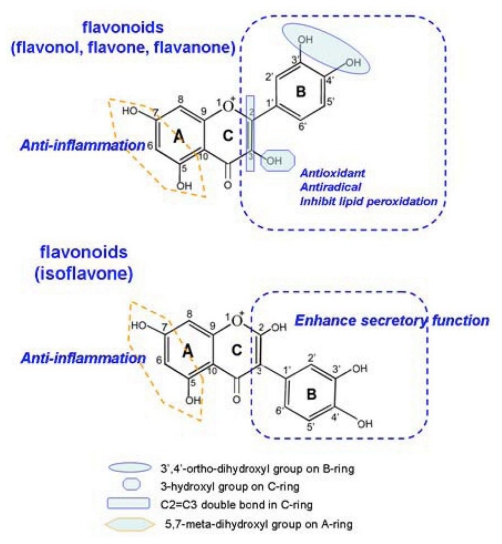
Schematic representation of the chemical requirements of 4-oxo-flavonoids (flavones, flavanones, flavonols and isoflavones) for inhibition of endothelial dysfunction.

In the present study, we analyzed the effect of 23 structurally related 4-oxo-flavonoids on oxLDL-induced endothelial dysfunction, an early event in the development of AS. We systematically analyzed the correlations between the structure characteristics of flavonoids and their different effects on oxidative stress-induced endothelial dysfunction. This study is one of very few that have used twenty-three representative 4-oxo-flavonoids categorized into four groups, flavones, isoflavones, flavanones and flavonols that are achievable following ingestion of flavonoids-rich foods, such as beans, fruits, vegetables and cereals. Generally, we have found that the inhibitory effects of 4-oxo-flavonoids on oxLDL-induced endothelial dysfunction were significantly correlated with the number of −OH in total and in B-ring. The presence of a 3′,4′-ortho-dihydroxyl on B-ring, a 3-hydroxyl on C-ring and a 2,3-double bond of C-ring appeared to be the main structural requirements for the antioxidant activity, while a 5,7-meta-dihydroxyl on A-ring was correlated to the anti-inflammatory capacity of 4-oxo-flavonoids. Isoflavones, which possess a B-ring substituted on C3 rather than C2 position of C-ring, could dominantly enhance NO release in endothelial cells, suggesting that the substituted position of B-ring might greatly influence the secretory function of endothelial cells (Figure 5). Additionally, hydroxylation at C6 position significantly attenuated the inhibitory effects of 4-oxo-flavonoids on endothelial dysfunction. This is the first report of the structural requirements of 4-oxo-flavonoids in inhibiting endothelial dysfunction induced by oxidative stress.

Recent studies emphasize the role of oxLDL in the pathogenesis of AS and showed that an elevated level of LDL is one of the most important risk factors for AS and cardiovascular morbidity [17,18]. Endothelial dysfunction elicited by oxLDL has been demonstrated as the key step in the initiation of AS, leading to lipid peroxidation (e.g., MDA production), apoptotic cell generation, adhesion molecules overexpression and reduction of the NO release. Several receptors of oxLDL have been identified so far, including SR-AI/II, CD36, SR-BI, FcγRII and LOX-1 expressed by endothelial cells. Among these receptors, LOX-1 is characterized as the major receptor for oxLDL in endothelial cells [19], and its inducible expression has been identified as a major cause of the endothelial dysfunction, leading to the up-regulation of genes related to apoptosis, inflammation, adhesion and secretion [20–22]. Increased expression of LOX-1 activates NADPH oxidase (subunit gp91phox), and increases the intracellular reactive oxygen species (ROS) generation. Excessive ROS directly results in lipid peroxidation (e.g., MDA generation), and is also associated with p38MAPK activation, NF-κB translocation as well as a proatherosclerotic, prooxidative, and proinflammatory gene expression pattern, together with accelerated AS [23]. The increase of intracellular ROS also attenuated the secretory function of endothelial cells, leading to the reduction of NO release.

Studies have clearly shown that flavonoids can enter into the circulation intact [24–28]. Flavonoids were mainly absorbed and excreted in intact forms and metabolized into methylated derivatives in human urine, although there is a rather low bioavailability [29]. Recently, it has been revealed that flavonoids could be incorporated into the plasma membrane and cytosol of endothelial cells and may be beneficial for scavenging ROS [30]. It is widely accepted that oxLDL-induced endothelial dysfunction is associated with an alteration of the cell redox status, thus the pre-incorporation of flavonoids by endothelial cells might enhance their resistance to the afterwards damaging effects resulting from ROS generation induced by oxLDL. Although there is very little knowledge about the different bio-absorption and bioavailability of different flavonoids inendothelial cells, it is supposed that the significant correlation between the inhibition of endothelial dysfunction and intracellular radical scavenging activity of flavonoids could be based upon flavonoids incorporation by endothelial cells. Thus, it is commonly assumed that flavonoids exert endothelium-protective potency through the antioxidant pathway. Many studies have focused on the association of −OH moieties with the antioxidant activity of flavonoids [31–33]. Previous studies demonstrated that hydroxyl groups on the B-ring play an important role in electron resonance and in electron donation to the oxidizing agent. This is consistent with our finding that the number of −OH in total or in B-ring was closely correlated with the inhibitory effects on endothelial dysfunction, suggesting that 4-oxo-flavonoids exerted the endothelium-protective potency partially through the antioxidant pathway. However, the antioxidatant potential of flavonoids is also affected by the position of −OH substitutions on the A, B or C-ring. Previous studies have reported that the torsion angle of B-ring with the rest of flavonoids molecule correlates with its overall scavenging activity [34,35]. Furthermore, the A-ring has poor reactivity toward peroxyl radicals, and it is not a significant contributor to the antiradical activity. Therefore, hydroxylation substitution on ring-B and C is important for the antioxidant and antiradical activity of flavonoids. Derivatization or substitution of this group probably affects the planarity of the molecule, causes B-ring to lose coplanarity to rings A and C, and reduces the potential of these compounds to chelate metal ions, thereby preventing the iron-induced peroxidation. Hence, our results showed that flavonols with 3′,4′-ortho-dihydroxyl and 3-hydroxyl group exerted stronger activity in promoting endothelial viability, decreasing lipid peroxidation and inhibiting cell apoptosis. Among the tested flavonols, myricetin and quercetin, showed stronger effect in attenuating the oxLDL-induced endothelial dysfunction, both of which possess a −OH on C3 of C-ring and two −OH moieties on C3′ and C4′ position of B-ring. Our results are also consistent with the published data [31].

It is widely recognized that the induction of endothelium-monocyte adhesion and inflammatory reaction strongly depends on the activation of the transcription factor, NF-κB [36]. Regulation of NF-κB is redox-sensitive due to the modification of critical cysteine residues in the upstream signal transducers (in particular protein kinases and phosphatases) and the transcription factors themselves, which may differentially affect their DNA binding capacity [37]. Extensive studies show that flavonoids may inhibit NF-κB and other redox-sensitive transcription factors and hence act as the anti-inflammatory agents [38–41]. Our results suggested that some flavonols, flavones and isoflavones, showed strong activity in preventing the inflammation molecules in endothelial cells, which might be resulted from the presence of 5,7-meta-dihydroxyl groups. This is consistent with the previous study of Lotito [42] that the A-ring 5,7-dihydroxyl groups but not the B-ring *per se* are required for flavonoids to inhibit the TNFα-induced adhesion molecules expression in endothelial cells. However, it remains unclear how 5,7-dihydroxyl groups was related to the inhibitory effects on endothelial inflammatory.

The generation of NO by endothelial nitric oxide synthase (eNOS) plays a major role in maintaining cardiovascular homeostasis by influencing blood pressure, endothelial dysfunction, vascular smooth muscle mitogenesis, matrix synthesis, leukocyte adhesion, and platelet aggregation. The expression of eNOS and secretion of NO have been shown to be unregulated by some flavonoids. There is some evidence that estrogen receptor (ER) is involved in signal transduction for eNOS expression. The interaction of estrogen with ER leads to the transcriptional activation of estrogen-responsive genes [43,44], including eNOS [45] and key antioxidant defense genes [46]. In addition to its genomic actions, estrogen rapidly stimulates eNOS activity in cultured endothelial cells. Isoflavones, such as genistein and daidzein, have also been demonstrated to be the natural ligands for ERs, particularly ERβ rather than ERα, which can elicit endothelium-dependent and -independent relaxation in arterial rings *in vitro* and *in vivo*. Previous studies have revealed that isoflavones are structurally similar to estrogen and generally bind to ERβ with higher affinity [47,48]. In our study, both genistein and daidzein showed significant effects on the NO release, which might result from the interaction of isoflavones and ER and the downstream regulation of eNOS expression. Therefore, the structural characteristic of a B-ring substituted on C3 of C-ring might help activate ER and then enhance the expression of eNOS and NO release.

## 5. Conclusions

In summary, our study revealed that 3′,4′-ortho-dihydroxyl, 3-hydroxyl, 2,3-double bond and 5,7-meta-dihydroxyl groups were the main structure requirements of 4-oxo-flavonoids for the protective potency against oxLDL-induced endothelial dysfunction. However, the potential roles of such characteristics in relation to the inhibitory effects on endothelial dysfunction might be differential. The results of the present study might provide some evidence for AS prevention and help to provide a basis for the design of potent antiatherosclerotic agents that will have therapeutic potential in the prevention of AS.

## Figures and Tables

**Table 1:** Molecular characteristics of 23 4-oxo-flavonoids tested in our study.

No.	Chemicals	Source	B-ring					C-ring		A-ring				2,3-double bond	The number of −OH			
			C2′	C3′	C4′	C5′	C6′	C2	C3	C5	C6	C7	C8		In total	A-ring	B-ring	C-ring
	**flavones:**																	
1	flavone	Sigma	−H	−H	−H	−H	−H	B-ring	−H	−H	−H	−H	−H	√	0	0	0	0
2	chrysin	Sigma	−H	−H	−H	−H	−H	B-ring	−H	−OH	−H	−OH	−H	√	2	2	0	0
3	apigenin	Sigma	−H	−H	−OH	−H	−H	B-ring	−H	−OH	−H	−OH	−H	√	3	2	1	0
4	luteolin	Sigma	−H	−OH	−OH	−H	−H	B-ring	−H	−OH	−H	−OH	−H	√	4	2	2	0
5	6-hydroxyflavone	Sigma	−H	−H	−H	−H	−H	B-ring	−H	−H	−OH	−H	−H	√	1	1	0	0
6	baicalein	Sigma	−H	−H	−H	−H	−H	B-ring	−H	−OH	−OH	−OH	−H	√	3	3	0	0
7	7-hydroxyflavone	Sigma	−H	−H	−H	−H	−H	B-ring	−H	−H	−H	−OH	−H	√	1	1	0	0
	**isoflavones:**																	
8	genistein	Sigma	−H	−H	−OH	−H	−H	−H	B−ring	−OH	−H	−OH	−H	√	3	2	1	0
9	daidzein	Sigma	−H	−H	−OH	−H	−H	−H	B−ring	−H	−H	−OH	−H	√	2	1	1	0
	**flavanones:**																	
10	hesperetin	Sigma	−H	−OH	−OCH_3_	−H	−H	B-ring	−H	−OH	−H	−OH	−H	−	3	2	1	0
11	flavanone	Sigma	−H	−H	−H	−H	−H	B-ring	−H	−H	−H	−H	−H	−	0	0	0	0
12	naringenin	Sigma	−H	−H	−OH	−H	−H	B-ring	−H	−OH	−H	−OH	−H	−	3	2	1	0
13	2′-hydroxyflavanone	Sigma	−OH	−H	−H	−H	−H	B-ring	−H	−H	−H	−H	−H	−	1	0	1	0
14	4′-hydroxyflavanone	Sigma	−H	−H	−OH	−H	−H	B-ring	−H	−H	−H	−H	−H	−	1	0	1	0
	**flavonols:**																	
15	galangin	Sigma	−H	−H	−H	−H	−H	B-ring	−OH	−OH	−H	−OH	−H	√	3	2	0	1
16	morin	Sigma	−OH	−H	−OH	−H	−H	B-ring	−OH	−OH	−H	−OH	−H	√	5	2	2	1
17	myricetin	Sigma	−H	−OH	−OH	−OH	−H	B-ring	−OH	−OH	−H	−OH	−H	√	6	2	3	1
18	fisetin	Sigma	−H	−OH	−OH	−H	−H	B-ring	−OH	−H	−H	−OH	−H	√	4	1	2	1
19	geraldol	Sigma	−H	−OCH_3_	−OH	−H	−H	B-ring	−OH	−H	−H	−OH	−H	√	3	1	1	1
20	quercetin	Sigma	−H	−OH	−OH	−H	−H	B-ring	−OH	−OH	−H	−OH	−H	√	5	2	2	1
21	gossypin	Sigma	−H	−OH	−OH	−H	−H	B-ring	−OH	−OH	−H	−OH	O-glucoside	√	5	2	2	1
22	3,6-dihydroxyflavone	Sigma	−H	−H	−H	−H	−H	B-ring	−OH	−H	−OH	−H	−H	√	2	1	0	1
23	3,7-dihydroxyflavone	Sigma	−H	−H	−H	−H	−H	B-ring	−OH	−H	−H	−OH	−H	√	2	1	0	1

**Table 2:** Effect of 4-oxo-flavonoids with different concentrations on oxLDL-induced endothelial viability. EA.hy926 cells were pretreated with different 4-oxo-flavonoids at a series of concentration (5, 10, 20, 40 and 80 μM) for 2 hs and exposed to oxLDL of 100 μg/mL for another 24 hs. The optical density was measured at 450 nm and the values were determined as the percentage of the control group.

Groups	Cell viability (% of control)					
		5 μM	10 μM	20 μM	40 μM	80 μM
Control	100.00					
0.2% DMSO	106.36 ± 10.32					
100 μg/mL oxLDL	47.11± 5.19 ##					
**flavones:**						
flavone		47.76 ± 4.77	48.27 ± 5.91	45.22 ± 5.50	45.88 ± 7.92	40.65 ± 6.53
chrysin		48.08 ± 3.83	47.29 ± 8.56	53.98± 5.64	58.73 ± 4.16	45.12 ± 2.94
apigenin		53.86 ± 2.89	50.67 ± 6.25	54.64± 8.36	48.73 ± 5.08	42.79 ± 5.35
luteolin		55.96 ± 1.99	59.48 ± 6.32	61.04 ± 6.74 *	68.27 ± 8.22 *	76.44 ± 4.46 **
6-hydroxyflavone		46.99 ± 7.92	43.90 ± 4.19	40.60 ± 6.04	35.34 ± 6.59	29.51 ± 4.84
baicalein		48.62 ± 2.28	50.25 ± 6.87	57.17± 6.38	65.81 ± 4.90 *	57.76 ± 5.51
7-hydroxyflavone		50.34 ± 3.31	49.12 ± 8.92	44.46 ± 9.43	40.47 ± 2.32	33.23 ± 3.36
**isoflavones:**						
genistein		51.15 ± 2.72	46.53 ± 6.70	52.81± 4.68	56.03 ± 7.71	43.46 ± 4.65
daidzein		47.88 ± 4.22	47.94 ± 8.29	46.85± 11.10	53.94 ± 6.72	35.38 ± 9.70
**flavanones:**						
hesperetin		49.72 ± 3.25	47.45 ± 3.03	57.32± 4.29	53.49 ± 2.89	45.89 ± 6.56
flavanone		44.69 ± 5.27	48.48 ± 6.40	42.94± 4.56	38.20 ± 5.77	34.37 ± 3.31
naringenin		49.47 ± 3.18	49.41 ± 7.63	54.10± 4.90	51.52 ± 6.26	40.41 ± 6.79
2′-hydroxyflavanone		49.21 ± 3.02	51.41 ± 6.40	44.29± 3.01	45.51 ± 11.98	41.70 ± 6.59
4′-hydroxyflavanone		51.64 ± 6.31	54.01 ± 5.54	49.50± 10.23	47.08 ± 3.38	43.23 ± 3.81
**flavonols:**						
galangin		52.15 ± 3.57	56.59 ± 3.56	62.29± 4.35 *	67.78 ± 5.69 *	68.56 ± 2.24 *
morin		53.24 ± 3.51	54.00 ± 7.73	59.72± 3.85	65.37 ± 11.06 *	71.60 ± 4.45 **
myricetin		53.07 ± 2.00	60.19 ± 5.12	68.41± 4.60 *	73.26 ± 3.61 **	82.58 ± 3.67 **
fisetin		51.66 ± 3.97	58.92 ± 3.44	64.49± 8.1 *	71.22 ± 4.73 **	79.40 ± 5.55 **
geraldol		49.35 ± 3.52	50.63 ± 7.49	45.57± 2.57	55.81 ± 2.60	40.24 ± 3.75
quercetin		50.75 ± 2.13	59.49 ± 7.71	66.32± 6.4 *	73.63 ± 3.87 **	81.53 ± 6.18 **
gossypin		48.99 ± 3.27	58.15 ± 3.34	65.31± 6.5 *	67.87 ± 4.50 *	78.78 ± 4.42 **
3,6- dihydroxyflavone		50.66 ± 6.11	50.11 ± 5.70	46.15± 7.99	39.38 ± 6.14	34.07 ± 4.31
3,7- dihydroxyflavone		45.74 ± 4.81	50.57 ± 7.01	42.87± 4.28	42.42 ± 4.67	39.12 ± 3.03

# *P* < 0.05,

## *P* < 0.01, compared with control group;

* *P* < 0.05,

** *P* < 0.01, compared with oxLDL group.

**Table 3:** Influence of changing substituents of 4-oxo-flavonoids on cell viability and the level of MDA, NO and sICAM-1 in oxLDL-induced EA.hy926 cells.

Paired comparison				Cell viability	MDA level	NO level	sICAM-1level
	A		B				
**(a)**	**B-ring**						
	flavanone		2′-hydroxyflavanone (2′-hydroxyl)	↑	↓	↓	↑
	morin(2′,4′-dihydroxyl)		quercetin(3′,4′-dihydroxy)	↑ *	↓ *	↑ *	↓ *
	geraldol (3′-methoxy, 4′-hydroxyl)		fistin (3′,4′-dihydroxyl)	↑ **	↓ *	↑ **	↓
	apigenin (4′-hydroxyl)		luteolin (3′,4′-dihydroxyl)	↑ **	↓ *	↓	↑
	3,7-dihydroxyflavone		fistin (3′,4′-dihydroxyl)	↑ **	↓ **	↑ **	↓ **
	flavanone		4′-hydroxyflavanone (4′-hydroxyl)	↑	↓ *	↑	↓
	chrysin		apigenin (4′-hydroxyl)	↓	↓ *	↑	↓ *
	chrysin		luteolin (3′,4′-dihydroxyl)	↑ *	↓ *	↑	↓ *
	galangin		morin (2′,4′-dihydroxyl)	↓	↓	↓	↓
	galangin		myricetin (3′,4′,5′-trihydroxyl)	↑ *	↓ **	↑ *	↓ **
	galangin		quercetin (3′,4′-dihydroxyl)	↑ *	↓ **	↑ *	↓ *
	morin (2′,4′-dihydroxyl)		myricetin (3′,4′,5′-trihydroxyl)	↑ *	↓ *	↑ *	↓ **
	morin (2′,4′-dihydroxyl)		quercetin (3′,4′-dihydroxyl)	↑ *	↓ *	↑ **	↓ **
	3,7-dihydroxyflavone		fistin (3′,4′-dihydroxyl)	↑ **	↓ **	↑ **	↓ *
	3,7-dihydroxyflavone		geraldol (3′-methoxyl, 4′-hydroxyl)	↑	↓*	↑	↓
	chrysin (2′,4′ - dihydroxyl)		luteolin (3′,4′-dihydroxyl)	↑ *	↓ **	↑	↓ *
	quercetin (3′,4′-dihydroxyl)		myricetin (3′,4′,5′-trihydroxyl)	↓	↓	↑	↑

**(b)**	**A-ring**						
	flavone		chrysin (5,7-dihydroxyl)	↑	↓	↑ **	↓
	flavone		6-hydroxyflavone (6-hydroxyl)	↓ *	↑ *	↓ *	↑ *
	flavone		baicalein (5,6,7-trihydroxyl)	↑ *	↑	↓ *	↓
	flavone		7-hydroxyflavone (7-hydroxyl)	↓	↓	↓	↑
	chrysin (5,7-dihydroxyl)		baicalein (5,6,7-trihydroxyl)	↑ *	↑ *	↓ **	↑
	chrysin (5,7-dihydroxyl)		7-hydroxyflavone (7-hydroxyl)	↓ *	↓	↓ **	↑ *
	chrysin (5,7-dihydroxyl)		6-hydroxyflavone (6-hydroxyl)	↓ **	↑ **	↓ **	↑ **
	genistein		daidzein	↓	↑	↓	↑ *
	fistin (7-hydroxyl)		quercetin(5,7-dihydroxyl)	↑	↓	↑	↓ **
	fistin (7-hydroxyl)		gossypin (5,7-dihydroxyl, 8-*O*-glucoside)	↓	↓	↓ **	↑
	6-hydroxyflavone (6- hydroxyl)		baicalein (5,6,7-trihydroxyl)	↑ **	↓	↓	↓*
	6-hydroxyflavone (6- hydroxyl)		7-hydroxyflavone (7-hydroxyl)	↑	↓ *	↓	↓ *
	quercetin (5,7- dihydroxyl)		gossypin (5,7-dihydroxyl, 8-*O*-glucoside)	↓	↑	↓ **	↑ **
	3,6-dihydroxyflavone (6-hydroxyl)		3,7-dihydroxyflavone (7-hydroxyl)	↑	↓	↑ *	↓ *

**(c)**	**C-ring**						
	chrysin		galangin (3-hydroxyl)	↑ *	↓	↑	↓ *
	luteolin		quercetin(3-hydroxyl)	↑	↓ *	↑ *	↓ **
	7-hydroxyflavone (7- hydroxyl)		3,7-dihydroxyflavone (3,7-dihydroxyl)	↑	↑	↑	↑

**(d)**	**2,3-double bond**						
	flavanone		flavone (2,3-double bond)	↑ *	↓	↓	↓ *
	naringenin		apigenin (2,3-double bond)	↓	↓ *	↑ *	↓ **

**(e)**	**Substituted position of B-ring**						
	apigenin (C2)		genistein (C3)	↑	↓	↑ *	↑ *

* *P* < 0.05,

** *P* < 0.01, A *vs.* B in paired comparison; ↑: A < B; ↓: A > B.
